# Effects of dietary protein levels on genes related to subcutaneous fat deposition and lipid metabolism in Tibetan sheep

**DOI:** 10.1186/s12864-025-11874-6

**Published:** 2025-07-16

**Authors:** Xianhua Zhang, Zhenling Wu, Jiacheng Gan, Rengeerli Sa, Wei Gao, Yu Zhang, Shengzhen Hou, Linsheng Gui

**Affiliations:** College of Agriculture and Animal Husbandry, Qinghai University, Xining, Qinghai China

**Keywords:** Tibetan sheep, Absolute quantitative lipidomics, Transcriptomics, Subcutaneous fat, Fatty acid

## Abstract

Subcutaneous fat deposition significantly influences animal growth, carcass quality, and meat characteristics. This study investigates the effects of varying dietary protein levels on backfat thickness, antioxidant capacity, fatty acid composition, differentially expressed genes (DEGs), and lipid molecules in Tibetan sheep. Sixty lambs were randomly assigned to two groups: a high-protein group (13.03% protein) and a low-protein group (11.58% protein), with each group containing 30 lambs (3 replicates per group, 10 lambs per replicate). Results showed that the low-protein group had significantly smaller fat cell diameters than the high-protein group (*P* < 0.05, as determined by H&E staining). Additionally, the low-protein group exhibited significantly higher activities of GSH-Px and SOD, and lower MDA content compared to the high-protein group. Gas chromatography identified 33 fatty acids in the fat samples, with oleic, stearic, and palmitic acids being most abundant. The LP group had significantly lower C22:0 and higher C20:2, C20:3n6, C20:4n6, and C20:3n3 levels than the HP group (*P* < 0.05). Transcriptomic analysis revealed 70 DEGs, of which 33 were upregulated and 37 were downregulated. KEGG analysis showed DEGs were enriched in 5 lipid metabolism pathways, including osteoclast differentiation, IL-17 signaling, and fluid shear stress/atherosclerosis. PPI analysis identified key lipid metabolism genes (FOS, FOSB, JUN, NR4A1, JUNB, PPARG). qRT-PCR validated RNA-Seq data accuracy. Lipid analysis detected 39 lipid classes and 2,605 lipid species, such as 856 TGs, 335 DGs, 279 Cer, 226 PCs, and 205 PEs. The LP group had higher DG and TG proportions, with significant increases in DG (40:4e), DG (32:1e), DG (34:4e), DG (20:5_18:2), and TG (16:18:1_18:3) levels. Correlation analysis showed that *NR4A1*, *FOS*, *JUN*, and *JUNB* positively correlated with catalase (CAT) activity, while *FOS*, *JUN*, and *JUNB* were linked to fatty acid metabolism and adipocyte development. *PPARG* positively correlated with PUFAs (C20:2, C20:3n6, C20:4n6, C20:3n3, and C20:5n3). Lipid differential molecules (DG (40:4e) and DG (20:5_18:2)) positively correlated with CAT activity, and DG (32:1e) positively correlated with C22:0. Lipid differential molecules including DG (40:4e), DG (32:1e), DG (34:4e), DG (20:5_18:2), and TG (16:18_18:3) negatively correlated with adipocyte diameter. In conclusion, a diet with 11.58% protein regulates lipid-related gene expression, enhances antioxidant capacity in subcutaneous fat, and increases unsaturated fatty acid content.

## Introduction

Tibetan sheep (*Ovis aries*), a crucial species in the livestock economy of the Tibetan Plateau, exhibits exceptional adaptability to the harsh conditions of high-altitude and low-oxygen environments [1]. As a cornerstone of the local pastoral economy, this species is not only a primary source of subsistence and income for indigenous herders but also plays a crucial role in maintaining the ecological balance of the plateau [2–4]. Tibetan sheep meat is well-known among consumers for its nutritional richness, tenderness, and distinctive aroma [5, 6]. In recent years, the Tibetan sheep industry has prioritized enhancing market competitiveness, improving product quality, optimizing breeding structures, and preserving the ecological environment of the Qinghai-Tibet Plateau. Consequently, breeding practices have transitioned from traditional grazing to more intensive methods [7–9]. During the fattening period, insufficient dietary protein can impair growth performance and hinder the deposition of unsaturated fatty acids (UFAs) in Tibetan sheep [10]. On the other hand, too much protein in the feed will lead to incomplete absorption of protein by Tibetan sheep, resulting in feed waste, thereby increasing nitrogen emissions, causing environmental pollution and hindering the sustainable development of Tibetan sheep animal husbandry [11].

Adipose tissue is a critical determinant of meat quality and growth performance in livestock and poultry [8]. Subcutaneous fat, predominantly located in the deeper layers of the skin, serves as a significant site for fat storage and plays a vital role in energy homeostasis and metabolic regulation in animals [12, 13]. Moreover, subcutaneous fat deposition is closely associated with reproductive performance and meat quality, making it an important economic trait in livestock and poultry production [14]. Furthermore, the fatty acids (FAs) composition enhances the organoleptic qualities of the meat, contributing to its unique flavor [15]. Diet composition is a key factor affecting the synthesis and metabolism of subcutaneous fat [16]. Elevated dietary protein has been shown to increase leptin levels in subcutaneous adipose tissue and downregulate SREBP-1c mRNA expression, thereby improving carcass quality [17]. For instance, a study on small-tailed Han sheep demonstrated that increased protein levels significantly enhanced rib fat thickness and intramuscular fat deposition efficiency [18]. These findings collectively suggest that modulating dietary protein levels can effectively influence fat deposition and carcass quality in livestock and poultry.

Few studies have used multi-omics approaches to assess the impact of dietary protein levels on fat metabolism in Tibetan sheep [19]. This study employs integrated transcriptomics and lipidomics approaches to investigate the impact of varying dietary protein levels on lipid regulatory mechanisms, antioxidant capacity, adipocyte development, and fatty acid (FA) composition in subcutaneous adipose tissue.

## Materials and methods

### Animals and diets

Sixty healthy Tibetan sheep with an initial average weight of 15.40 ± 0.81 kg (2 months) were randomly selected from the Jinzang sheep breeding demonstration base (Haiyan, Qinghai Province, China). The lambs were randomly assigned into two groups, each consisting of 30 lambs (with 3 replicates per group, 10 lambs per replicate). The two groups of lambs were fed diets with protein levels of 11.58% (low protein, LP) and 13.03% (high protein, HP), to compare the effects of dietary protein levels on lamb growth and health [20]. The diet consisted of 70% concentrates and 30% roughage (oat hay: oat silage = 1:1), The nutritional requirements of lambs were calculated according to the Nutrient requirement of meat-type sheep and goat (NY/T 816–2021),with the detailed composition and nutritional content provided in Table 1.

**Table 1:** Ingredients and nutritional composition of the diets (dry matter based) %

Items	Group	
	LP	HP
Ingredient (%)		
Oat hay	15.00	15.00
Oat silage	15.00	15.00
Corn	32.20	32.41
Wheat	7.70	4.90
Soybean meal	1.40	5.60
Rapeseed meal	11.20	11.20
Cottonseed meal	0.70	1.40
Maize germ meal	0.70	1.40
Palm meal	11.20	8.40
NaCl	0.68	0.62
Limestone	0.70	0.70
Baking soda	0.07	0.07
Premix^1^	2.94	2.94
Lys	0.39	0.29
Met	0.13	0.07
Total	100.00	100.00
Nutrient levels^2^		
Digestibility/MJ·kg^-1^	9.61	9.61
Crude protein	11.58	13.03
Ether extract	3.36	3.36
Neutral detergent fiber	33.03	32.95
Acid detergent fiber	15.43	15.25
Ca	0.99	1.10
P	0.57	0.60

^1^ The premix provided the following per kg of diets: Cu 18 mg, Fe 66 mg, Zn 30 mg, Mn 48 mg, Se 0.36 mg, I 0.6 mg, Co 0.24 mg, VA 24 000 IU, VD 4 800 IU, VE 48 IU

^2^ Digestible energy is calculated and the rest are measured

The diets were prepared fresh twice a day and were offered as total mixed ration(concentrate: forage ratio of 7:3)in two equal meals at 8:00 am and 17:00 pm. Before the experiment, the pens and exercise areas were disinfected, cleaned, and the stalls were secured. Routine immunization with vaccines and parasite control measures were conducted for the lambs.

### Sample collection and backfat thickness determination

At the end of the experiment, 3 sheep were randomly selected from 3 replicates per group, respectively. At the end of the experiment, 3 sheep were randomly selected from 3 replicates per group, which were euthanized according to the ARRIVE guidelines (American Veterinary Medical Association Guidelines for Animal Euthanasia: 2020 edition) to slaughter. Pentobarbital sodium 80 to 100 mg/kg was injected into the jugular vein to ensure smooth delivery of the drug into the sheep. Pentobarbital Sodium was injected intravenously into the jugular vein at a dose of 80–100 mg/kg to ensure the smooth administration of the drug into the sheep’s body. After the injection, the sheep usually lost consciousness within 30 s to 1 min and died within 3–5 min. They were then slaughtered after confirming their death (by absence of corneal reflex, auscultation for no heartbeat, and fixed and dilated pupils). The subcutaneous adipose tissue of longissimus dorsi muscle was collected as experimental material [21]. A portion of the tissue was fixed in paraformaldehyde solution for subsequent morphometric analysis, while another portion was rapidly frozen in liquid nitrogen and stored at −80 °C for subsequent transcriptomic and lipid metabolic analyses.At the same time, the thickness of the backfat was measured from the subcutaneous fat (12 − 13 th ribs) of the carcass using a vernier caliper [22]. Tissue thickness was measured at 11 cm from the midline of the spine, between the 12th and 13th ribs, using a vernier caliper [23].

### Hematoxylin & Eosin (H&E) staining

Subcutaneous adipose tissue samples were fixed in 4% neutral formaldehyde for at least 48 h, embedded in paraffin, and sectioned. The sections were deparaffinized, stained with H.E., dehydrated, sealed, and evaluated using light microscopy. Adipocyte diameter and area were measured with slide viewer (SV) analysis software (Jinan Tangier Electronics Co., Ltd., China) [24].

### Enzyme linked immunosorbent assay (Elisa)

The antioxidant indexes, including catalase (CAT), superoxide dismutase (SOD), total antioxidant capacity (T-AOC), glutathione peroxidase (GSH-Px), and malondialdehyde (MDA), were determined by ELISA (Jiangsu Meibiao biotechnology co., Ltd.). Briefly, 0.5 g of subcutaneous fat was homogenized in 900 µL phosphate-buffered saline (PBS, Helen, Changsha, China), centrifuged at 3,000 × g for 20 min at 4 °C, and the supernatant was collected for testing

### FA composition determination

FAs were separated by gas chromatography on an Agilent DB-FastFAME capillary column (20 m × 0.18 mm I.D. × 0.2 μm). The gas chromatography conditions were as follows: initial temperature 80 °C, held for 30 s; ramped at 70 °C/min to 175 °C, and 8 °C/min to 230 °C for 1 min. After reaching 230 °C, held for 2 min. A quality control (QC) sample was included with each experimental sample to assess system stability and performance. Mass spectrometry conditions: electron impact ionization (EI), ion source temperature 230 °C, quadrupole temperature 150 °C, transmission line temperature 240 °C, electron energy 70 eV. Ion scanning was performed in selected ion monitoring (SIM) mode.

### Ranscriptomic analyses

Total RNA was extracted from subcutaneous adipose tissue using an RNA extraction kit (Invitrogen, Carlsbad, CA, USA) in accordance with the manufacturer’s protocol. RNA integrity was assessed using an Agilent Bioanalyzer 4150 (Agilent Technologies, CA, USA) and a NanoDrop ND-2000 (Thermo Scientific, USA). Libraries were prepared following the manufacturer’s protocol. After cDNA validation and quantification, the libraries were sequenced on the Illumina HiSeqTM 4000 platform, with Clean Reads mapped to the Ovis aries (Oar_v3.1) reference genome using the HISAT2 software and a modified BWT algorithm.

Differential gene expression was analyzed using DESeq2 [25] to identify differentially expressed genes (DEGs) between subcutaneous fat from low- and high-protein groups. The P less than 0.05 and| log2 (foldchange)| greater than 1 was used as the criteria for screening the DEGs. The function of each DEGs is to classify the DEGs according to their biological functions through gene ontology (GO) enrichment and Kyoto Encyclopedia of Genes and Genomes (KEGG) pathway enrichment analysis [26].

To further explore the biological relationships between genes, we conducted a protein-protein interaction (PPI) network analysis using the STRING database. The generated PPI files were imported into the CytoNCA plugin in Cytoscape 3.9.1 to identify hub genes with high scores using the betweenness centrality algorithm.

### Absolute quantitative lipidomics

MS-grade methanol, acetonitrile, and HPLC-grade 2-propanol (Thermo Fisher), along with HPLC-grade formic acid and ammonium formate (Sigma), were used. Samples were spiked with internal lipid standards and homogenized with water and methanol. MTBE was added, and the mixture was sonicated at 4 °C for 20 min before being incubated at room temperature for 30 min. The solution was centrifuged, and the upper organic solvent layer was evaporated under nitrogen. Lipid analysis was performed using a liquid chromatography-tandem mass spectrometry (LC-MS/MS) system with a CSH C18 column for separation. Lipid extracts were re-dissolved, centrifuged, and 3 µL of the sample was injected. Solvent A consisted of acetonitrile-water with formic acid and ammonium formate. Solvent B was a mixture of acetonitrile-isopropanol with formic acid and ammonium formate. The initial mobile phase was 30% solvent B, which was increased linearly to 100% over 23 min and equilibrated at 5% for 10 min. Mass spectra were acquired using a Q-Exactive Plus in both positive and negative ion modes. ESI parameters were as follows: source temperature, 300 °C; capillary temperature, 350 °C; ion spray voltage, 3000 V; S-Lens RF level, 50%; scan range, m/z 200–1800.

### Real-Time quantitative PCR (qRT-PCR) analysis

Six genes were randomly selected for qRT-PCR analysis to validate the accuracy of the transcriptome sequencing data. Total RNA isolated from subcutaneous adipose tissue was reverse-transcribed into cDNA using the PrimeScriptTM RT Reagent Kit (TaKaRa, China). qRT-PCR was conducted using the SYBR^®^TremexExTaq™ Kit (TaKaRa, China) with three biological replicates. The 2^−ΔΔCt^ method was employed to calculate the relative gene expression levels for each mRNA. The primer sequences were designed using an online primer design tool (Bioengineering Co., Ltd., Shanghai, China) (Table 2).

**Table 2:** The primer sequence design list

Name	Primer sequence(5’−3’)	Tm(℃)	Product length
*FOS*	TCTTCCTTCGTCTTCACCTACCC	60.0	81 bp
	GTTGCTGCTGCTGCCCTTG	60.0	
*EGR1*	CAGGAGTGATGAACGCAAGAGG	60.0	95 bp
	GAGAGGAGGTGGCAGCAGAG	60.0	
*JUN*	CCAAGAACTCCGACCTCCTCAC	60.0	97 bp
	TGCCCGTTGCTGGACTGTATG	60.0	
*JUNB*	CCACGACGAATCATACGGAACAG	60.0	85 bp
	GCTGGGTTTCAGGAGTTTGTAGTC	60.0	
*NR4A1*	CAGCCTCCTCCACATCCTCATC	60.0	100 bp
	CTCAGCGTGCCAGGGTAGC	60.0	
*PPARG*	GAGCCTGCGAAAGCCCTTTG	60.0	80 bp
	CATCTAATTCCAGTGCGTTGAACTTC	60.0	
*GAPDH*	ACCTGCCGCCTGGAGAAAC	60.0	104 bp
	TGGTCCTCAGTGTAGCCTAGAATG	60.0	

### Data processing and analysis

Data were analyzed using SPSS 26.0 software to compare the two groups. Data are expressed as the mean ± standard error of the mean (SEM). The independent samples t-test was performed to assess statistical significance, followed by the Bonferroni post hoc test; *P* < 0.05 was considered statistically significant. Pearson correlation analysis was conducted to examine the relationships between lipid differential molecules, differential genes, and phenotypic traits.

## Results

### H&E staining results with backfat thickness

**Fig. 1:**
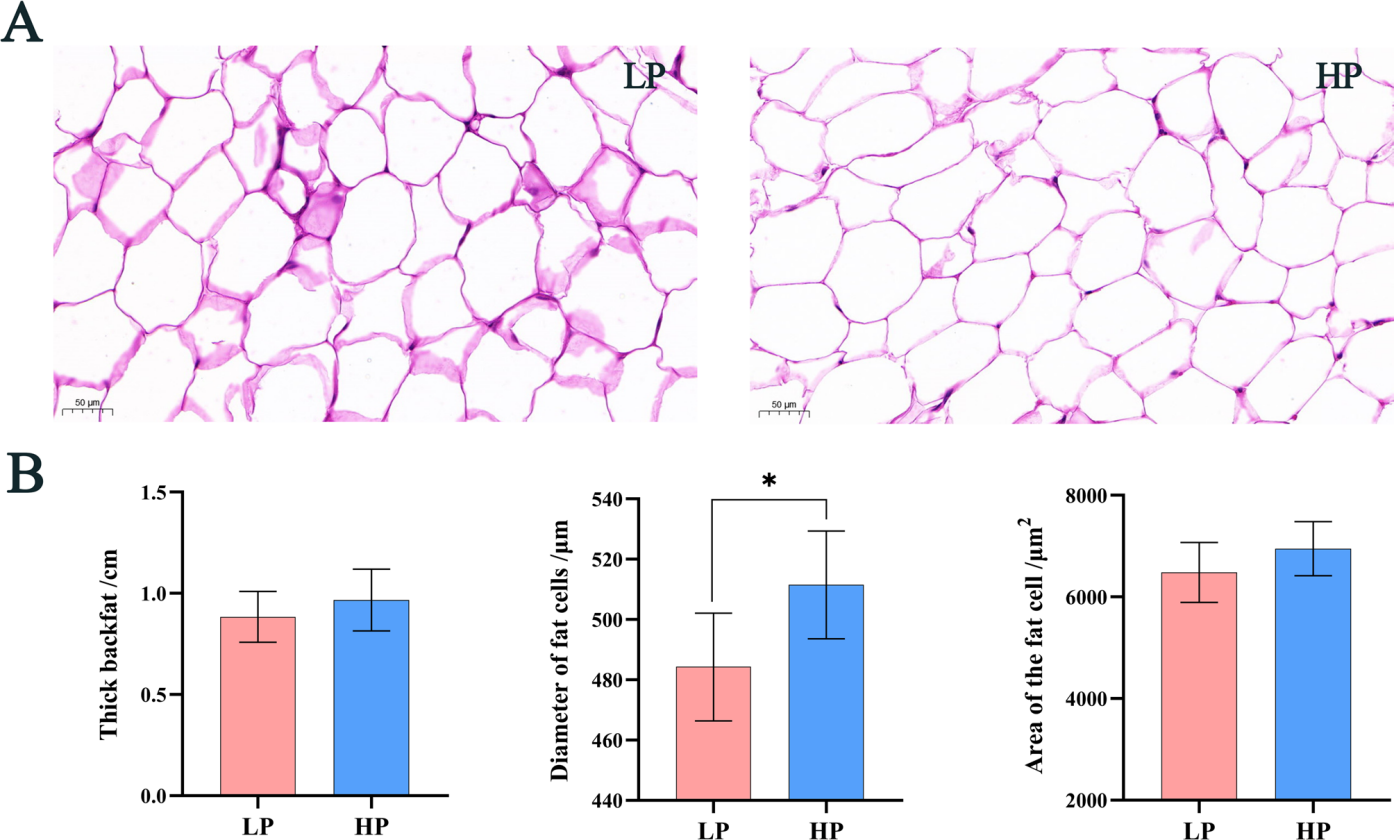
**A **Subcutaneous fat stained sections. **B** Diameter, area and backfat thickness of subcutaneous fat. The magnification is 20x. In the histogram, * represents *p* < 0.05

The H&E staining results are shown in Fig. 1A, revealing that the subcutaneous adipocytes of Tibetan sheep are large, well-defined, and predominantly round, oval, or multilobular. Furthermore, as shown in Fig. 1B, no significant difference in backfat thickness was observed between the HP and LP groups (*P* > 0.05). However, the diameter of adipocytes in the HP group was significantly larger than that in the LP group (*P* < 0.05).

### Antioxidant capacity

**Fig. 2:**
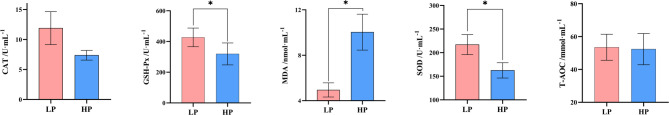
Effects of diet with high and low protein levels on the antioxidant capacity of subcutaneous fat. * *p* < 0.05

As depicted in Fig. 2, the activities of GSH-Px and SOD in the LP group were significantly higher than those in the HP group (*P* < 0.05). In contrast, the MDA content in the LP group was significantly lower than that in the HP group (*P* < 0.05). No significant differences were observed in T-AOC and CAT activity between the HP and LP groups (*P* > 0.05).

### Determination of FAs in subcutaneous adipose tissue

A total of 33 FAs were identified in this study, with oleic acid (C18:1n9c), stearic acid (C18:0), and palmitic acid (C16:0) being the predominant FAs. As shown in Table 3, among saturated fatty acids (SFA), the content of behenic acid (C22:0) in the LP group was significantly lower than in the HP group (*P* < 0.05). Among polyunsaturated fatty acids (PUFAs), the contents of eicosatrienoic acid (C20:3n6), arachidonic acid (C20:4n6), eicosatetraenoic acid (C20:3n3), and C20:2 were significantly higher in the LP group than in the HP group (*P* < 0.05).(Table 3).

**Table 3:** Comparison of fatty acid content between the two groups %

Item	Group		SEM	*P*-value
	LP	HP		
Saturated fatty acids(SFA)				
C6:0	0.02	-	-	-
C8:0	0.07	0.05	0.02	0.136
C10:0	0.84	0.814	0.02	0.295
C11:0	0.02	0.08	0.06	0.087
C12:0	3.12	1.84	0.45	0.181
C13:0	0.19	0.12	0.07	0.051
C14:0	39.35	38.02	5.16	0.281
C15:0	4.40	4.59	0.69	0.320
C16:0	169.17	186.54	22.42	0.268
C17:0	9.63	13.24	1.37	0.478
C18:0	163.75	181.59	22.75	0.275
C20:0	3.64	2.32	0.69	0.143
C21:0	0.17	0.14	0.06	0.075
C22:0	0.27^b^	0.50^a^	0.13	0.034
C23:0	0.16	0.15	0.02	0.073
C24:0	0.18	0.15	0.04	0.331
Monounsaturated fatty acid				
C14:1	1.11	1.13	0.20	0.106
C15: 1	0.32	0.31	0.10	0.148
C16: 1	15.65	17.43	2.29	0.272
C17: 1	4.38	5.82	0.71	0.315
C18:1n9c	256.87	277.48	34.67	0.212
C20:1n9	8.63	6.78	1.30	0.354
C22:1n9	5.22	3.81	0.80	0.205
Polyunsaturated fatty acid				
C18:2n6c	25.22	15.69	10.82	0.079
C18:2n6t	3.08	2.60	0.24	0.131
C18:3n6	0.24	0.19	0.05	0.235
C18:3n3	2.58	2.20	0.47	0.079
C20:2	0.66^a^	0.55^b^	0.06	0.023
C20:3n6	0.34^a^	0.24^b^	0.11	0.024
C20:4n6	0.74^a^	0.52^b^	0.26	0.018
C20:3n3	0.17^a^	0.11^b^	0.07	0.020
C20:5n3	0.13	0.07	0.13	0.074
C22:2n6	0.26	0.13	0.07	0.079

Independent samples were statistically significant at *P* 0.05

### RNA-seq and mapping

Table 4 presents the basic statistics for the RNA sequencing of subcutaneous fat in Tibetan sheep. A total of 38.52 million raw reads were obtained after sequencing six samples. In this sequencing, 95.39–98.40% of the clean reads aligned with the Tibetan sheep reference genome, far exceeding the typical threshold of 70%. The unique mapping rate ranged from 56.79 to 61.84%, while the multiple mapping rate varied between 35.38% and 40.41%. The results indicate that 13.49–18.01% of the spliced reads were mapped to the genome, while 39.98–46.90% of the unspliced reads aligned with the genome.

**Table 4:** Difference analysis of RNA SEQ data

Sample	Total reads(M)	Total mapped	Multiple mapped	Unique mapped	Non-splice reads	Splice reads
LP-1	69.88	97.88%	37.81%	60.06%	43.21%	16.85%
LP-2	73.48	98.40%	40.41%	57.99%	39.98%	18.01%
LP-3	48.76	97.34%	37.59%	59.75%	43.42%	16.33%
HP-1	66.32	97.22%	35.38%	61.84%	46.90%	14.94%

### Analysis of gene expression

**Fig. 3:**
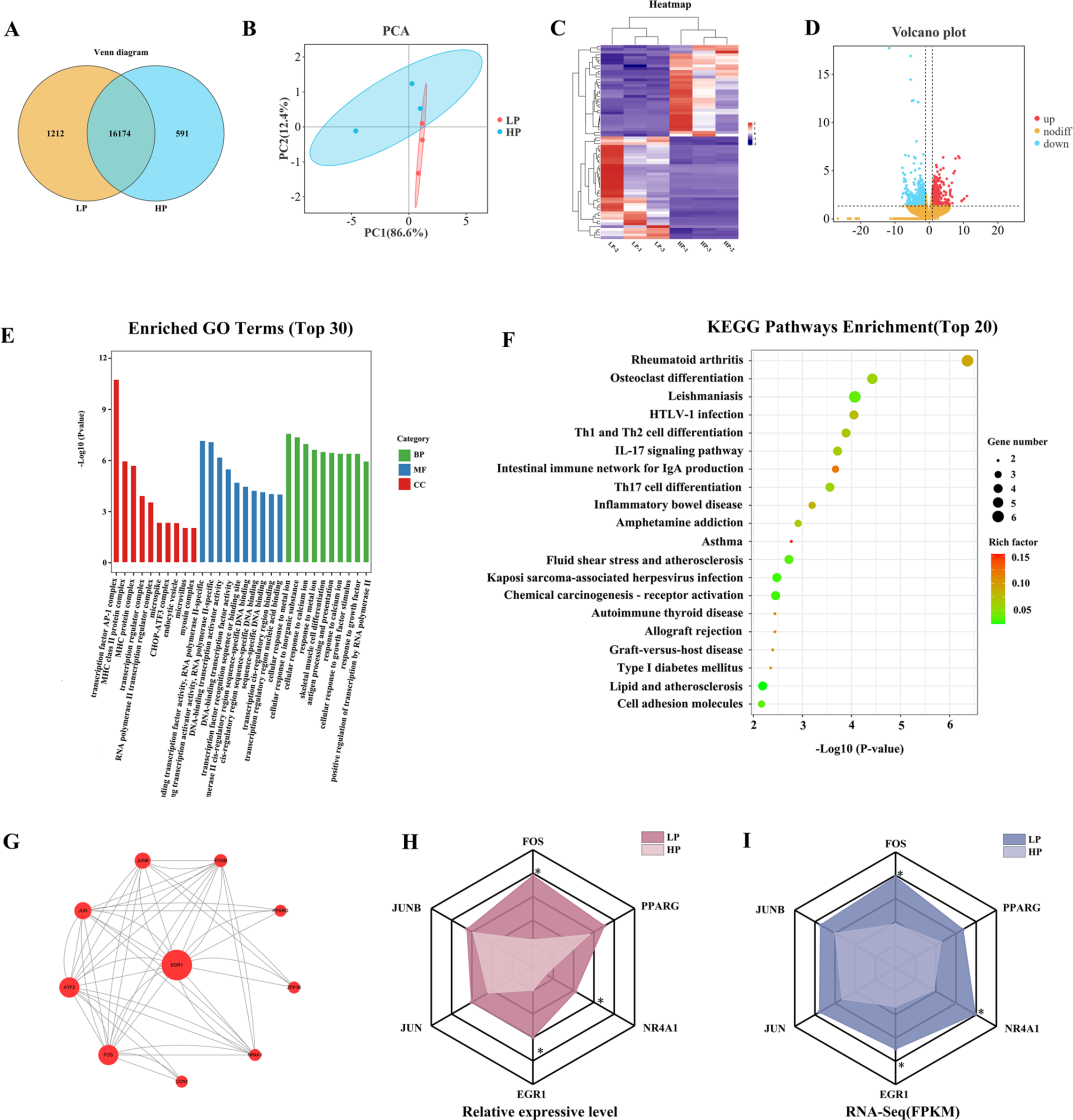
Transcriptomic analysis of subcutaneous fat under LP group and HP group (**A**) Venn diagram; **B** PCA diagram; **C** Heatmap analysis of differentially expressed genes; **D** Volcano map; **E** KEGG analysis of differentially expressed genes; **F** GO analysis of differentially expressed genes. Note: The ordinate is the *P* value after -log10 processing, and the entries are arranged in ascending order from the largest to the lowest *P*-value. From left to right: red represents cellular components (CC), blue molecular function (MF), green biological processes (BP), and abscissa is the description of specific functions; **G** PPI gene network map; **H** Radargram of differentially expressed genes based on qRT-PCR; **I** Radargram of differentially expressed genes based on FPKM values

The Venn diagram illustrates that 17,386 and 16,765 DEGs were identified, with 16,174 DEGs in the intersection (Fig. 3A). The two principal components of PCA explained 86.6% and 12.4% of the variance, with the first two components accounting for 99% of the total variation. PCA revealed a significant separation between the two groups (Fig. 3B). A clustering analysis of the differential genes was conducted in this study (Fig. 3C), where the color gradient from blue to red represents an increase in gene expression levels from low to high. The samples used in this study exhibited clear clustering, suggesting their suitability for further analysis. A total of 70 DEGs were identified between the two groups based on the Q-value (< 0.05), with 37 upregulated and 33 downregulated (Fig. 3D).

### Function enrichment analysis of DEGs

To explore the functional associations of the DEGs, GO and KEGG enrichment analyses were performed using the appropriate annotation databases. Between the LP and HP groups, 407 GO terms were found to be significant (*P* < 0.05) across three major functional categories: biological processes (BP, 328), cellular components (CC, 19), and molecular functions (MF, 60). The top 10 functional entries, sorted by P-value, under the three major GO categories are presented as bar graphs (Fig. 3E).

A total of 70 DEGs (FDR < 0.05, *P* < 0.05) were detected between the two groups, with 33 DEGs upregulated and 37 DEGs downregulated. KEGG pathway enrichment analysis identified five signaling pathways related to cell proliferation, differentiation, immune response, and lipid metabolism, including osteoclast differentiation, Th1 and Th2 cell differentiation, Th17 cell differentiation, IL-17 signaling pathway, and fluid shear stress (Fig. 3F). PPI analysis identified potential key genes associated with lipid metabolism, including *FOS*, *FOSB*, *JUN*, *NR4A1*, *JUNB*, and *PPARG* (Fig. 3G).

To verify the accuracy of the transcriptome sequencing results, RT-qPCR was performed on six DEGs related to lipid metabolism, including *FOS*, *JUN*, *NR4A1*, *JUNB*, *EGR1*, and *PPARG* (Fig. 3H-I). The results indicated that the upregulation and downregulation trends in gene expression were consistent with the RNA-seq data, confirming the reliability of the transcriptome analysis.

### Lipidomic analysis

**Fig. 4:**
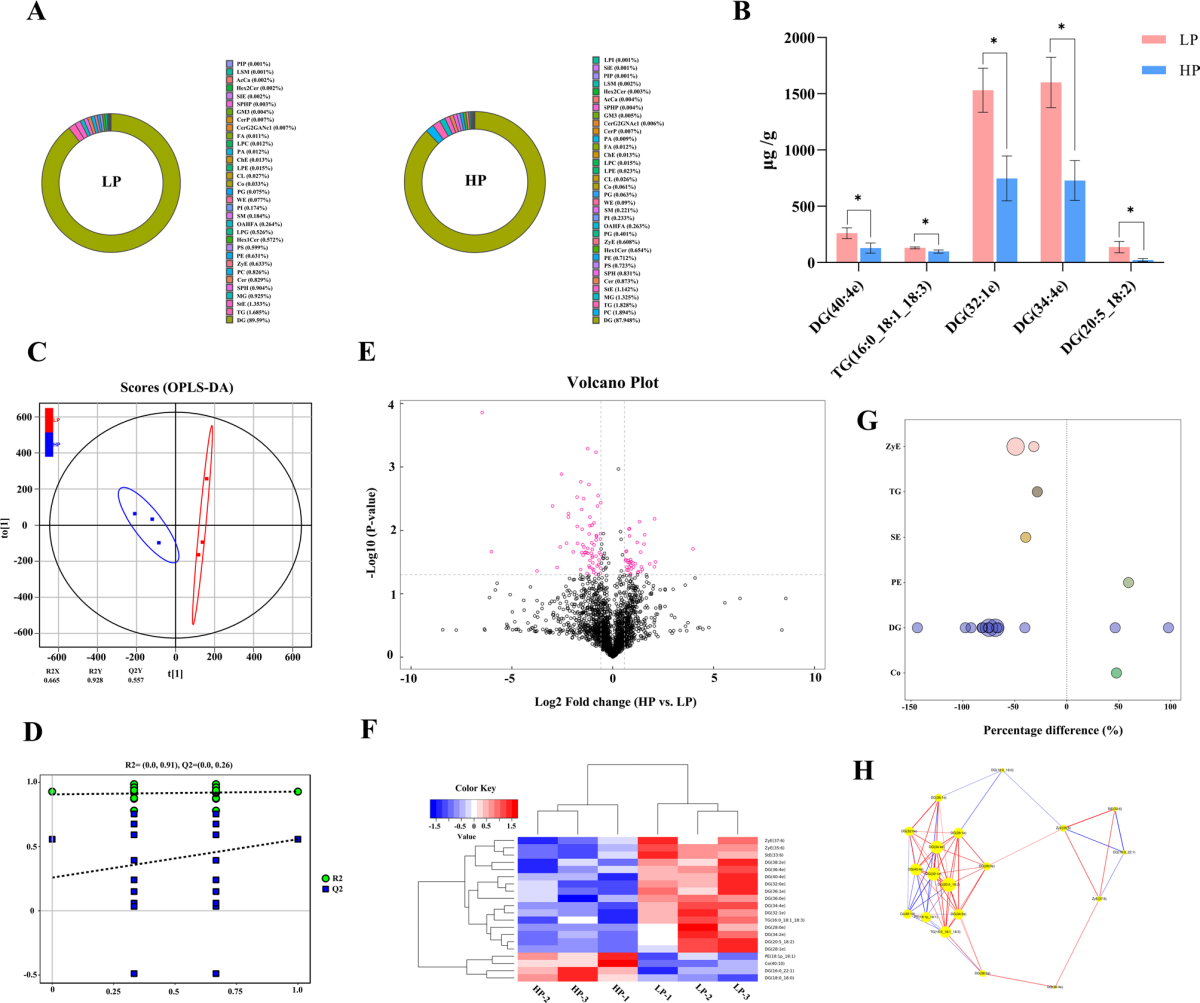
LP group and HP group Lipidomics analysis of the subcutaneous fat samples.** A **Composition of lipid subclasses; **B** Bar chart of differentially expressed lipid molecules; **C** Orthogonal partial least squares discrimination analysis (OPLS-A of lipids ) score chart; **D** OPLS-DA permutation test; **E **Volcano map of differential lipid molecules; **F** Heat map analysis of differential lipid molecules; **G** Descriptive statistics of differential lipid metabolites; **H **Differential lipid molecular network Figure

Diliracylglycerol (DG), triacylglycerol (TG), and stigmasteryl ester (StE) were the lipid subclasses comprising more than 1% in the LP group, while DG, phosphatidylcholine (PC), and TG were predominant in the HP group (Fig. 4A). The projected importance of variables values (VIP, VIP > 1) and P-values (*P* < 0.05) obtained from the Orthogonal partial least squares discriminant (OPLS-DA) model were used to identify lipid species that differed between the LP and HP samples(Figure 4B). The OPLS-DA plot demonstrated clear separation between the LP and HP samples (Fig. 4C-D). Based on Ultra high performance liquid chromatography-tandem mass spectrometry (UHPLC-MS/MS) analysis, 39 lipids and 2,605 lipid species were identified, including 856 triglycerides (TGs), 335 ditriglycerides (DGs), 279 ceramides (Cer), 226 phosphatidylcholine (PCs), 205 phosphatidylethanolamine (PEs), and other lipid classes. The majority of lipids in both groups were dilacylglycerols (DG) (Fig. 4E). Hierarchical clustering of samples from each group was performed using the expression levels of significant differential lipids (VIP > 1, *P* < 0.05), resulting in clear clustering suitable for further analysis (Fig. 4F).

Sixteen lipids (12 DGs, 2 ZyEs, 1 TG, 1 StE) were significantly more abundant in the LP group compared to the HP group. These included DG (40:4e), StE (33:6), DG (28:0e), DG (28:1e), TG (16:0_18:1_18:3), DG (32:0e), DG (32:1e), ZyE (35:6), ZyE (37:6), DG (34:2e), DG (34:4e), DG (36:4e), DG (36:0e), DG (36:1e), DG (36:4e), DG (38:2e), and DG (20:5_18:2), all of which were significantly upregulated. The remaining 4 lipids (2 DGs, 1 CoQ10, 1 PE) were significantly lower in the LP group than in the HP group, including DG (16:0_22:1), DG (18:0_18:0), CoQ10, and PE (18:1p_18:1), which were significantly downregulated. Bubble plots were used to display significantly altered lipid molecules (Fig. 4G). Correlation analysis was performed to assess the metabolic proximity between significant differential lipids (VIP > 1, *P* < 0.05) and to further elucidate their mutual regulatory relationships during biological state transitions. The significant differential molecular network analysis revealed a strong positive correlation between DG (40:4e), TG (16:0_18:1_18:3), DG (32:1e), DG (34:4e), and DG (20:5_18:2, Fig. 4H).

### Correlation analysis of phenotype, transcriptomics and lipidomics

**Fig. 5:**
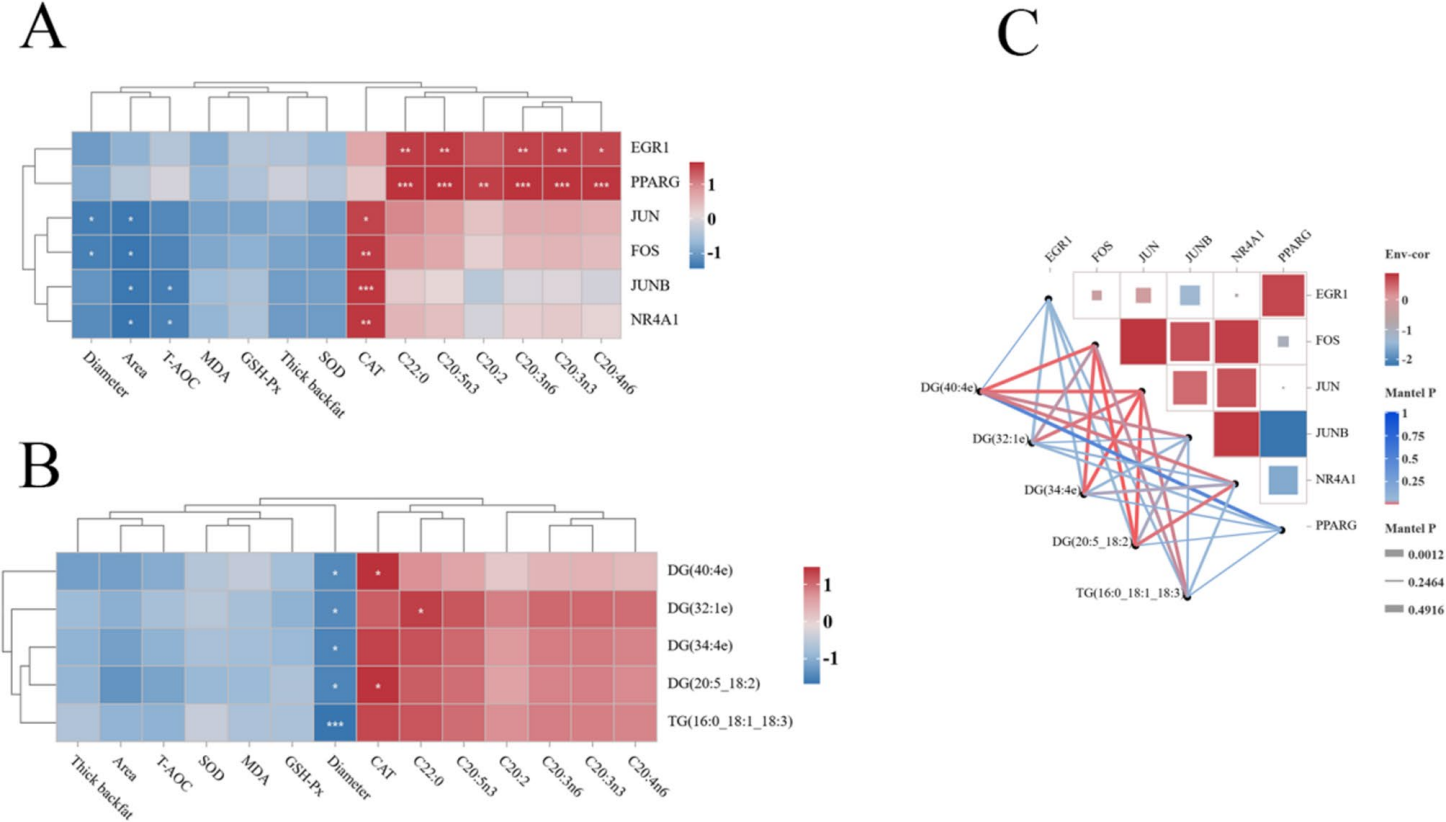
Correlation analysis of phenotype, transcriptomics and lipidomics: **A **Association analysis of differential genes and phenotypes; **B **Association analysis of differential lipid molecules and phenotype; **C** Association analysis of differential genes and differential lipid molecules

Association analysis of differential genes and phenotypic traits, including antioxidant capacity, sebum thickness, FAs, and adipocyte diameter and area, revealed that *EGR1* and *PPARG* were positively correlated with C20:2, C20:3n6, C20:4n6, C20:3n3, C20:5n3 and C22:0. *JUN*, *JUNB*, *FOS* and *NR4A*1 genes were positively correlated with CAT. *JUN* and *FOS* were negatively correlated with adipocyte diameter and area, and *JUNB* and *NR4A1* were negatively correlated with T-AOC and adipocyte area (Fig. 5A). Correlation analysis of differential lipid molecules and phenotypic traits, including antioxidant activity, backfat thickness, FAs, and adipocyte diameter and area, demonstrated that DG (40:4e), TG (16:0_18:1_18:3), DG (32:1e), DG (34:4e) and DG (20:5_18:2) were negatively correlated with adipocyte diameter. DG (32:1e) was positively correlated with C22:0. DG (40:4e) and DG (20:5_18:2) were positively correlated with CAT (Fig. 5B). Association analysis of DEGs and differential lipid molecules revealed that the *JUN* and *FOS* genes were positively correlated with lipid molecules such as DG (40:4e), TG (16:0_18:1_18:3), DG (32:1e), DG (34:4e), and DG (20:5_18:2). The *PPARG* gene exhibited an inverse correlation with the lipid molecule DG (40:4e) (Fig. 5C).

## Discussion

The fat content of sheep carcasses is influenced by factors such as the composition of dietary nutrients (protein, fat, and energy), breed, environment, and the duration of feeding [27–30]. Tibetan sheep in the Qinghai-Tibet Plateau are capable of surviving the dry grass period (November to May), withstanding the severe cold of the plateau and adapting to the low-oxygen environment, which is closely associated with their subcutaneous fat deposition. Studies have demonstrated that fat deposition in livestock is directly proportional to the size of adipocytes [31], reflecting the increased efficiency of high-protein diets in promoting fat deposition. The results of this study indicated that both the diameter of subcutaneous adipocytes increased with higher dietary protein levels. These findings are consistent with previous results [32]. Therefore, when the energy levels in the diet are controlled, a high-protein diet leads to an increase in the volume of subcutaneous adipocytes in Tibetan sheep. Based on previous studies, it can be inferred that under a high-protein diet, the rumen produces more volatile fatty acids, which are subsequently stored as triglycerides in adipocytes, leading to their enlargement [33]. Although protein levels in the diet can affect the diameter of adipocytes, they may reduce the efficiency of subcutaneous fat deposition, and further research is needed to understand how to regulate this process.

Redox homeostasis is essential for maintaining normal physiological functions in mammals [34]. After slaughter, redox processes in livestock and poultry continue, resulting in the production of large amounts of reactive oxygen species (ROS) [35]. Research has shown that excessive accumulation of ROS can induce apoptosis, leading to the oxidation of UFAs in fat, which reduces its water-holding capacity and negatively affects meat quality [36]. Enzymatic antioxidants, including SOD, CAT, and GSH-Px, are crucial for scavenging free radicals and maintaining cellular integrity [37]. In this study, dietary protein levels did not affect the enzymatic activities of SOD and CAT; however, GSH-Px activity was significantly higher in the low-protein group compared to the high-protein group, suggesting that the low-protein group had an enhanced ability to scavenge lipid peroxides. Furthermore, the MDA content in subcutaneous fat was significantly lower in the low-protein group compared to the high-protein group, supporting the previous finding. Some studies have shown that MDA content reflects the extent of lipid peroxidation, and that GSH-Px catalyzes the conversion of reduced glutathione to its oxidized form, facilitating the removal of lipid peroxides [38–40].

FAs play a critical role in various biological processes and system regulation, including immune responses, enzyme activity, cell proliferation and differentiation, as well as the regulation of gene expression [41–43]. Research by Francisco et al. [44] has demonstrated that the content and composition of FAs in the subcutaneous fat of Tibetan sheep could influence meat quality to varying extents. In this study, a total of 16 SFAs, 7 MUFAs, and 10 PUFAs were identified in subcutaneous fat samples from Tibetan sheep in both low and high protein groups, with higher FA content correlating with improved meat quality [45]. The study found that SFAs (C22:0) increased with higher protein levels, consistent with the findings of Tous et al. [46]. It is hypothesized that when protein intake is high, proteins are broken down into amino acids, undergo deamination, and the resulting carbon skeletons are subsequently converted into precursors for FA synthesis [47]. Additionally, the concentrations of C20:3n6, C20:4n6, C20:3n3, and C20:5n3 increased with decreasing protein levels in this experiment. These findings are consistent with those of Catellani et al. [48]. As previously mentioned, low protein levels enhance the antioxidant capacity of the subcutaneous adipose tissue in Tibetan sheep. Due to their reactive nature, UFAs are prone to reacting with free radicals, leading to lipid peroxidation. Furthermore, UFAs and pro-antioxidants work synergistically in subcutaneous fat [49, 50]. It is speculated that this may explain the higher content of UFAs in the low-protein group.

This study utilized RNA-Seq technology to investigate the impact of varying protein levels on the expression of genes related to subcutaneous fat in Tibetan sheep. The results indicated that the DEGs were predominantly enriched in pathways related to cell proliferation and differentiation, immune response, and lipid metabolism. During adipocyte proliferation and differentiation, fibroblast-like cells accumulate nutrients, ultimately differentiating into mature adipocytes that are filled with triglycerides [51]. The FA metabolism pathway plays a crucial role in subcutaneous fat deposition, with the synthesis, degradation, and transport of FAs directly influencing subcutaneous fat content [52]. These findings suggest that these pathways are primarily enriched in FA metabolism and physiological processes associated with subcutaneous fat deposition, with nutrient regulation influencing both subcutaneous fat accumulation and nutrient utilization in ruminants [53].

In this study, RNA sequencing (RNA-Seq) was employed to investigate the impact of varying protein levels on the expression of subcutaneous fat-related genes in Tibetan sheep. The results indicated that DEGs were primarily enriched in pathways related to cell proliferation and differentiation, immune response, and lipid metabolism. During the proliferation and differentiation of adipocytes, fibroblast-like cells initially accumulate nutrients, which then form mature adipocytes that are filled with triglycerides [54]. The FA metabolism pathway plays a critical role in subcutaneous fat deposition, where the synthesis, breakdown, and transport of FAs directly influence the amount of subcutaneous fat [55]. This study demonstrated that varying dietary protein levels influenced the expression of genes involved in the proliferation and differentiation of subcutaneous adipocytes in Tibetan sheep. *NR4A1* expression promotes insulin secretion, inhibits the differentiation and maturation of adipocytes, exerts anti-adipogenic effects, and subsequently impacts glucose and lipid metabolism [56]. Intracellular redox balance is disrupted during glycolipid metabolism. The study by Palumbo-Zerr et al. demonstrated that NR4A1-/- knockout mice exhibited disturbances in glucose and lipid metabolism, with fat deposition occurring in the liver after high-fat feeding, accompanied by increased oxidative stress levels [57]. Normal *NR4A1* expression contributes to maintaining appropriate intracellular lipid levels, reduces ROS from lipid peroxidation, thereby alleviating oxidative stress and preventing the depletion of antioxidant enzymes such as catalase (CAT), potentially modulating CAT activity positively [58]. *NR4A1* was positively correlated with CAT, indicating that the upregulation of *NR4A1* in response to a low-protein diet promotes the regulation of CAT activity and reduces lipid peroxidation. In addition, the *FOS*, *JUN*, and *JUNB* genes play crucial roles in adipocyte proliferation, differentiation, and FA metabolism [59–61]. *JUNB* is predominantly expressed in brown adipose tissue, where it functions as a transcriptional regulator, integrating signals to inhibit related pathways by binding to the ERRα promoter. It regulates the transformation and energy metabolism of thermogenic adipocytes and influences fat deposition [62]. At the cellular level, *JUNB* has been shown to interact with C/EBPβ to inhibit adipocyte differentiation [63]. The present study found that the expression of *NR4A1*, *FOS*, *JUN*, and *JUNB* gene was significantly up-regulated, and these four genes were negatively correlated with adipocyte area, indicating that low protein levels can effectively inhibit the proliferation and differentiation of adipocytes and promote fat deposition. Additionally, *FOS*, *JUN*, and *JUNB* influence plasmalogen synthesis by modulating citric acid metabolism or signaling pathways, regulating acetyl-CoA carboxylase activity, or binding to transcription factors, thereby promoting FA synthesis [64]. However, *FOS*, *JUN* and *JUNB* were not shown to promote FA synthesis in this experiment, and further studies are needed. During subcutaneous fat deposition, both the stress state of adipose tissue and the inflammatory response influence adipocyte metabolic function [53]. Under conditions of stress or inflammation, changes in DEG expression can alter FA metabolism in adipose tissue [65]. The inflammatory response induces the release of specific FAs from adipocytes to meet the body’s energy or immunomodulatory demands during stress, thereby indirectly influencing subcutaneous fat deposition [66].

The peroxisome proliferator-activated receptor (PPAR) signaling pathway is a crucial transcriptional regulator involved in lipid metabolism and the inflammatory response. It promotes FA oxidation, reduces lipid accumulation, and mitigates ROS production. Additionally, it inhibits the expression of inflammatory factors, thereby reducing oxidative stress [67]. *PPARG* is a member of the PPAR gene subfamily, encoding nuclear receptors [68]. During fat metabolism, activation of *PPARG* enhances FA oxidation, promotes mitochondrial β-oxidation, decreases plasma free FA levels, and reduces both fat accumulation and insulin resistance [69]. Reactive oxygen species (ROS) production is closely linked to fat metabolism, with excessive fat accumulation contributing to increased oxidative stress [70, 71]. *PPARG* modulates the expression of fatty acid desaturase 2 (FADS2) and fatty acid elongation enzyme 5 (ELOVL5), thereby influencing the biosynthesis of long-chain PUFAs in fish. FADS2 and ELOVL5 are key enzymes in long-chain polyunsaturated fatty acid synthesis. FADS2 introduces double bonds into the fatty acid carbon chain, while ELOVL5 is responsible for elongating the chain. *PPARG* regulation of these enzymes influences the synthesis of long-chain polyunsaturated fatty acids [72, 73]. Correlation analysis revealed a significant positive correlation between *PPARG* and PUFAs (C20:3n3, C20:3n6, C20:4n6, C20:5n3). These findings are consistent with those reported by Zhang et al. [74]. These findings suggest that *PPARG* influences the cellular redox state through its regulation of fat metabolism, with *PPARG* activation promoting the expression of antioxidant genes and increasing related enzyme activities.

Lipidomics, a subfield of metabolomics, employs LC-MS/MS to precisely quantify lipid molecules, thus enabling the exploration of the relationship between lipid molecules and phenotypic traits [75]. Lipid function studies typically emphasize changes at the subclass level, as lipids often exert their functions within subclass groups, making it challenging to delineate the roles of individual lipid molecules within the same subclass. Subcutaneous fat deposition in animals represents a complex biological process regulated by multiple factors [76]. The lipid subclasses examined in this study, along with FAs and catalase (CAT) antioxidant capacity, play a pivotal role in this process, interacting to form a complex regulatory network. FAs serve as crucial precursors for the synthesis of triglycerides (TG) in animals, with diacylglycerol (DG) acting as an intermediate product in TG synthesis [77]. Research in nutritional metabolism has demonstrated that excess energy and protein intake is converted and stored through complex metabolic pathways when it surpasses the body’s immediate energy requirements [78]. Excess energy can drive the participation of substances such as glucose and amino acids in the tricarboxylic acid cycle through intermediate metabolites like acetyl-CoA [79]. At the cellular level, FAs are taken up by adipocytes, activated by fatty acyl-CoA synthetase, and progressively esterified with glycerol-3-phosphate to form triglycerides (TG) [80]. During this process, an increase in lipid subclasses may promote esterification reactions, thereby driving subcutaneous fat deposition [81]. In this study, the molecular levels of DG and TG increased as dietary protein levels decreased, suggesting a close relationship between FA synthesis and enhanced esterification. As fat deposition progresses in adipocytes, fat catabolism generates substantial reactive oxygen species (ROS), and ROS accumulation may result in cellular damage, thereby affecting fat metabolism and deposition [82]. Concurrently, we observed a positive correlation between lipid molecules (DG (40:4e), DG (20:5_18:2)) and catalase (CAT). Lipid molecules synergistically regulate subcutaneous fat deposition, collaborating to modulate FA metabolism and antioxidant capacity [83]. The results of this trial indicate that a low-protein diet may enhance subcutaneous fat deposition.

Diacylglycerol (DG) content increased as dietary protein levels decreased. The increase in DG serves as a signaling molecule that regulates lipid composition and distribution on cell membranes, thereby affecting the activity of phosphatidylinositol 3-kinase (PI3K) and its ability to bind to cell membranes [84]. The activation of PI3K catalyzes the production of PIP3 (phosphatidylinositol-3,4,5-triphosphate), which then acts as a second messenger to activate Akt (protein kinase B) [85]. Akt activation promotes cell survival, proliferation, and metabolism. In adipocytes, Akt regulates the expression of genes related to lipid synthesis and metabolism, thus influencing lipid accumulation. Akt can also phosphorylate specific transcription factors, which indirectly regulate the expression of genes such as *FOS* and *JUN*, thereby establishing a positive feedback loop that promotes subcutaneous fat deposition [86, 87]. The lipid molecular network influences the activity of signaling pathways where DEGs are located, by regulating the lipid microenvironment within cells. Activation of the ERK signaling pathway upregulates the expression of lipid synthesis genes, including FAS (fatty acid synthase) and acetyl-CoA carboxylase (ACC), promoting FA synthesis and triglyceride accumulation [88]. Simultaneously, the ERK signaling pathway regulates the expression and activity of lipid transporters, including lipoprotein lipase (LPL), thus promoting lipid uptake and storage [89]. In contrast, activation of the p38 MAPK and JNK signaling pathways upregulates the expression of lipolysis-related genes and promotes lipid catabolism in adipocytes [90]. It subsequently regulates the differentiation, proliferation, and expression of genes related to lipid metabolism in adipocytes, ultimately influencing the amount of subcutaneous fat deposition. Therefore, a low-protein diet contributes to an increased proportion of lipid molecules, promoting lipid antioxidant activity and adipocyte differentiation.

## Conclusion

In summary, this study demonstrated that dietary protein levels significantly influence the antioxidant capacity, fatty acid (FA) content, gene expression, and lipid composition of subcutaneous fat in Tibetan sheep. Specifically, a dietary protein level of 11.58% was found to enhance antioxidant capacity and increase the content of unsaturated fatty acids (UFAs) in subcutaneous fat. Integrated RNA-sequencing and lipidomics analyses identified several key genes associated with lipid metabolism, including *FOS*, *FOSB*, *JUN*, *NR4A1*, *JUNB*, and *PPARG*. Additionally, pathways related to osteoclast differentiation, Th1 and Th2 cell differentiation, Th17 cell differentiation, the IL-17 signaling pathway, fluid shear stress, and atherosclerosis were implicated. These findings provide critical insights into the metabolic regulation of subcutaneous fat in Tibetan sheep and offer a theoretical basis for the development of optimized dietary strategies to improve meat quality and overall animal performance.

## Data Availability

The dataset used in this paper is available from the authors (15009702909@163.com). The data reported in this paper have been deposited in the OMIX, China National Center for Bioinformation / Beijing Institute of Genomics, Chinese Academy of Sciences (https://ngdc.cncb.ac.cn/omix: accession no.OMIX010636). Transcriptome data were deposited in NCBI SAR (accession: PRJNA1278963).

## Abbreviations

H&E: Hematoxylin & eosin
CAT: Catalase
GSH-PX: Glutathione peroxidase
SOD: Superoxide dismutase
T-AOC: Total antioxidant capacity
MDA: Malondialdehyde
FAs: Fatty acids
UFAs: Unsaturated fatty acids
PUFAs: Polyunsaturated fatty acids
SV: Slide viewer
DEGs: Differentially expressed genes
PPI: Protein-protein interaction
DG: Diliracylglycerol
TG: Triacylglycerol
StE: Stigmasteryl ester
